# Supplementary material for: Drug repurposing for aging research using model organisms
Source: Aging Cell. 2017 Jun 16;16(5):1006–15. doi: 10.1111/acel.12626 (PMC5595691; doi:10.1111/acel.12626)
Supplement: Supplementary file 7 — Data S1 Zip‐Archive of all report cards. [file ACEL-16-1006-s007.zip › RC_SIN.pdf]

SIN

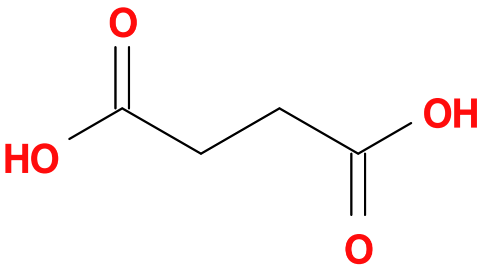

Database identifiers

ChEMBLCompound	CHEMBL576
DrugBank	DB00139
CHEBI	15741
eMolecules	481572

Ranking

	Rank	Score
Drosophila	NA	NA
C. elegans	338/591	0.176

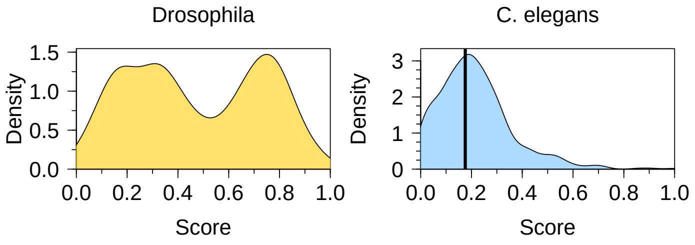

	Ageing implication	Domain conservation	Binding site conservation	Binding affinity	Bioavailability	Lipinski	Promiscuity	Purchasability	Drug approval	**Total**
Drosophila	NA	NA	NA	NA	NA	NA	NA	NA	NA	NA
C. elegans	0.36	0.138	0.426	0.216	0.219	0.0	-0.0	0.1	0.075	0.176

Names

- **succinic acid**
- 1,2-ethanedicarboxylic acid
- acide butanedioique
- acide succinique
- acidum succinicum
- amber acid
- asuccin
- Bernsteinsaeure
- Butandisaeure
- Butanedionic acid
- Dihydrofumaric acid
- E363
- Ethylenesuccinic acid
- Hooc-ch2-ch2-cooh

· spirit of amber

Roles

micronutrient, radiation protective agent, anti-ulcer drug, fundamental metabolite, nutraceutical

Status

Approved drug (according to ChEMBL)	No
Classification (according to DrugBank)	approved, nutraceutical
Administration Route	Oral
Number of Rule of 5 violations	0
Binding affinity to original target in log units (RF-Score prediction)	3.71
Burns *C. elegans* bioavailability prediction	-4.34

Medical Information

Indicatation: For nutritional supplementation, also for treating dietary shortage or imbalance
Mechanism of action: Succinate is an essential component of the Krebs or citric acid cycle and serves an electron donor in the production of fumaric acid and FADH2. It also has been shown to be a good "natural" antibiotic because of its relative acidic or caustic nature (high concentrations can even cause burns). Succinate supplements have been shown to help reduce the effects of hangovers by activating the degradation of acetaldehyde - a toxic byproduct of alcohol metabolism - into CO2 and H2O through aerobic metabolism. Succinic acid has been shown to stimulate neural system recovery and bolster the immune system. Claims have also been made that it boosts awareness, concentration and reflexes.
Toxicity: Oral rat LD50: 2260 mg/kg
Retail: Breckenridge Pharmaceuticals, Contract Pharm, J T Baker, KV Pharmaceutical Co., Murfreesboro Pharmaceutical Nursing Supply, National Vitamin Company and 6 more suppliers
Succinic acid cryst reagent - 0.15 USD / g

(Information from DrugBank)

Compound Target Characteristics

Fibronectin

Best gene implication in ageing for this target family came from gene Q99KD0 via mapping the annotation from Ensembl ENSMUSG00000026193 via mapping the annotation from EntrezGene 14268 via mapping the annotation from GenAgeModels 0818 annotated in GenAge release 17.

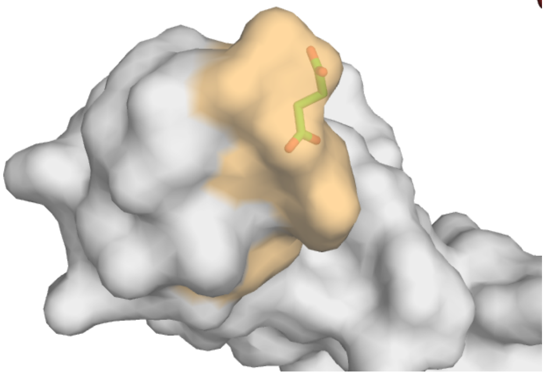

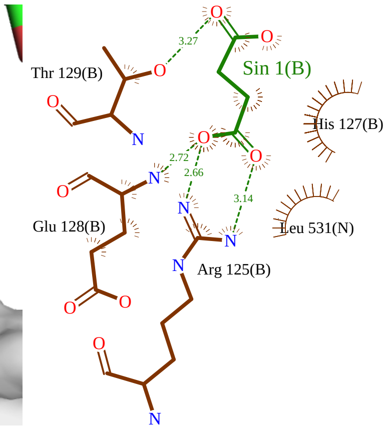

protein	amino acids contacts (binding site)
PDB:2rkz:chainA:P02751	R H E T
tr:Q53S27:Q53S27_HUMAN	R G E N
tr:Q9H382:Q9H382_HUMAN	R G E N
tr:Q7L553:Q7L553_HUMAN	R G E N
tr:H0Y7Z1:H0Y7Z1_HUMAN	R G E N
tr:F8W7G7:F8W7G7_HUMAN	R G E N
sp:P02751:FINC_HUMAN	R G E N
tr:F1LST1:F1LST1_RAT	R G E N
tr:Q99KD0:Q99KD0_MOUSE	R G E N
tr:Q3UZF9:Q3UZF9_MOUSE	R G E N
sp:P11276:FINC_MOUSE	R G E N
tr:H2KYR5:H2KYR5_CAEEL	G P Y T
tr:Q18914:Q18914_CAEEL	G P Y T

	whole protein		domain-based		contact-based	
protein	ident	simil	ident	simil	ident	simil
PDB:2rkz:chainA:P02751	0.4	0.77	0.55	0.84	1.0	1.0
tr:Q53S27:Q53S27_HUMAN	0.06	0.06	1.0	1.0	0.5	0.62
tr:Q9H382:Q9H382_HUMAN	0.07	0.07	1.0	1.0	0.5	0.62
tr:Q7L553:Q7L553_HUMAN	0.09	0.09	1.0	1.0	0.5	0.62
tr:H0Y7Z1:H0Y7Z1_HUMAN	0.44	0.46	1.0	1.0	0.5	0.62
tr:F8W7G7:F8W7G7_HUMAN	0.93	0.93	1.0	1.0	0.5	0.62
sp:P02751:FINC_HUMAN	1.0	1.0	1.0	1.0	0.5	0.62
tr:F1LST1:F1LST1_RAT	0.65	0.69	0.97	0.99	0.5	0.62
tr:Q99KD0:Q99KD0_MOUSE	0.08	0.09	0.97	0.99	0.5	0.62
tr:Q3UZF9:Q3UZF9_MOUSE	0.4	0.43	0.97	0.99	0.5	0.62
sp:P11276:FINC_MOUSE	0.88	0.94	0.97	0.99	0.5	0.62
tr:H2KYR5:H2KYR5_CAEEL	0.03	0.12	0.07	0.42	0.25	0.43
tr:Q18914:Q18914_CAEEL	0.03	0.12	0.07	0.42	0.25	0.43
